# The standalone aminopeptidase PepN catalyzes the maturation of blasticidin S from leucylblasticidin S

**DOI:** 10.1038/srep17641

**Published:** 2015-12-01

**Authors:** Guiyang Yu, Li Li, Xiangyang Liu, Guang Liu, Zixin Deng, Mark T. Zabriskie, Ming Jiang, Xinyi He

**Affiliations:** 1 State Key Laboratory of Microbial Metabolism and School of Life Science and Biotechnology, Shanghai Jiao Tong University, Shanghai 200030 (China); 2 Engineering Research Center of Industrial Microbiology (Ministry of Education), and College of Life Sciences, Fujian Normal University, Fuzhou, Fujian 350108 (China); 3 Department of Pharmaceutical Sciences, College of Pharmacy, Oregon State University, Corvallis, OR 97331-3507 (USA)

## Abstract

The peptidyl nucleoside blasticidin S (BS) isolated from *Streptomyces griseochromogenes* was the first non-mercurial fungicide used on a large scale to prevent rice blast. In the biosynthesis of BS, leucylblasticidin S (LBS) was suggested as the penultimate metabolite with 20-fold less inhibitory activity than the final product BS. Incomplete conversion of LBS to BS at a variable efficiency ranging from 10% to 90% was observed either in the native strain *S. griseochromogenes* or a heterologous producer *Streptomyces lividans* WJ2. In this study, we determined that maturation of BS from LBS is not a spontaneous process but is governed by a standalone peptidase PepN, which hydrolyzes LBS in a pH-sensitive way with most appropriate of pH 7~8 but is inactive when the pH is below 5 or above 10. PepN1 and PepN2, two neighboring PepN homologs from *Streptomyces lividans* were purified in *E. coli* but displayed ca.100-fold difference in LBS hydrolytic activity. Overexpression of *pepN1* in WJ2 enhanced BS yield by 100% and lowered the ratio of LBS to BS from 2:1 to 2:3. This work presents the expansion of the biological role for PepN in antibiotic maturation and the first report of hydrolysis of beta amide linkage by this conserved enzyme.

Blasticidin S (BS) is a nucleoside analog consisting of a cytosine linked to a dideoxyhexose and bonded to a modified arginine. BS was isolated from the broth of *Streptomyces griseochromogenes* in an effort to identify natural non-mercurial fungicides^1^. BS has been used on a large scale to control rice blast by foliar application against the virulent fungus (*Pyricularia oryzae*) which was the cause of the disease in Asia^2^. BS was found to strongly inhibit the production of aflatoxin by *Aspergillus flavus* without impairing fungal growth^3^.

Blasticidin S exerts its inhibitory activity by interfering with ribosomal protein synthesis in host cells. Unlike other peptidyltransferase inhibitors, such as clinically used chloramphenicol, linezolid, the lincosamides lincomycin and clindamycin, which bind to the A site of the large ribosomal subunit^4^,^5^,^6^, BS occupies the P site, where it induces conformational changes of the P-site tRNA^7^.

As BS displayed non-selective toxicity to prokaryotic and eukaryotic cells, it is of great interest to understand the resistance mechanism, particularly the mechanism for self-resistance. There have been two groups of BS resistance genes reported in non-BS producers. The first group featured by BSD or BSR encodes blasticidin S deaminase^8^,^9^,^10^,^11^,^12^ that converts BS to non-toxic deaminohydroxyblasticidin S. This group of resistance genes along with BS are widely used in the selection of the transformed cells, particularly in eukaryotic transgenic studies^13^,^14^,^15^. The other group is blasticidin S acetyltransferase that uses acetyl-coenzyme A to acetylate the amino group at C-13 of blasticidin S to give N-acetylblasticidin S^16^,^17^,^18^. In some fungi, two groups of resistance genes are present to convert BS to N-acetyldeaminohydroxyblasticidin S^3^. Impermeability of the cell membrane constitutes another barrier to delivery of BS to its intracellular targets. But some nutrient uptake systems could be hijacked for BS delivery, such as an ABC transporter named NppA1A2BCD^19^ and the leucine-rich repeat-containing 8(LRRC8) proteins^20^ in mammalian cells.

In studies of blasticidin S, demethylblasticidin S (DBS)^21^ and leucylblasticidin S (LBS)^22^ were isolated as the intermediates in the broth of *S. griseochromogenes* when the pH of the culture medium was kept below 4. However, LBS was later isolated from *Streptomyces* sp. SCC 1785 and *S. griseochromogenes* under the normal laboratory conditions^23^,^24^. Addition of leucine to C-13 of the beta-amino group of BS was expected as a new self-resistance mechanism because the antibiotic activity of LBS against indicator strain *Bacillus circulans* was decreased by 20 folds compared to BS^23^. LBS was suggested as the direct precursor for BS biosynthesis as the washed cells^22^ or cell lysate^23^ of *S. griseochromogenes* can convert LBS into BS (Fig. 1A). It is of particular interest to know where this conversion occurs to avoid self-destruction of the host by the generated BS, as well as identifying the protein responsible for this hydrolysis *in vivo*.

In this study, we determined that PepN from *E. coli* and *Streptomyces lividans* can catalyze the hydrolysis of LBS into BS. The activity of PepN is pH-dependent wherein it shows the highest hydrolytic activity at pH 7–8 and loses its activity when pH is below 5 or above 10. The variation in the ratio between LBS to BS in the fermentation broth of *S. lividans* is attributed to the changes in activity of PepN that is affected by the medium pH in different fermentations.

## Results

### LBS and BS productivity is significantly affected by fermentation conditions

The blasticidin S native producer *Streptomyces griseochromogenes* has a very strong barrier system against introduction of foreign DNA. Heterologous expression of the BS biosynthetic gene cluster in *Streptomyces lividans* initially only yielded LBS, but not BS^25^. As the published BS gene cluster contains nine putatively unrelated genes upstream of *blsS* but stops at *blsN*, immediately downstream of essential gene *blsM*, we speculated that there might exist a peptidase gene downstream of *blsM* and thus cloned 8780-bp sequences (Accession number is JX244070) after *blsN* through chromosomal walking. We engrafted the expanded BS biosynthetic gene cluster onto *S. lividans* to generate WJ2, which is able to produce BS^11^. We then conjectured that the 8780-bp sequence might encode the putative LBS peptidase gene.

**Figure 1:**
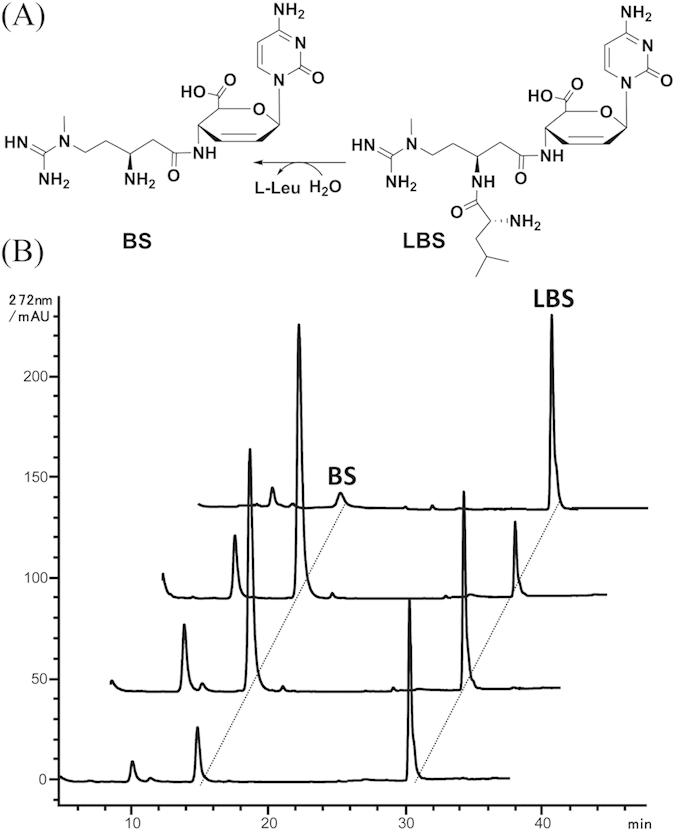
Transformation of LBS to BS is significantly affected by fermentation conditions. (**A)** Schematic representation of transformation of LBS to BS; (**B)** HPLC analysis of metabolites produced by *S. lividans* WJ2 which was engrafted with the expanded BS biosynthetic gene cluster in different fermentations. BS is abbreviated for the blasticidin S, LBS is for leucylblasticidin S. The ratio between LBS to BS varied from 2:9 to 9:1 in different fermentations.

*blsN*, *bls-1* and *bls-2* were then independently knocked out in *S. lividans* WJ2, and analysis of the metabolites and biological activity assay of three mutants revealed no difference from strain WJ2 (Supplementary Figure S1A,B), implying they are not peptidase genes or there exist multiple peptidase gene(s) elsewhere. Unexpectedly, we observed that all these mutants as well as WJ2 can produce LBS in addition to BS. The ratio between LBS to BS varied from 2:9 to 9:1 in different fermentations (Fig. 1B). It therefore raised two possibilities with respect to the maturation of BS from LBS: i) a LBS hydrolase gene locates outside of BS gene cluster and its activity is significantly affected by the fermentation condition; ii) LBS hydrolysis is a spontaneous process that is greatly affected by fermentation conditions.

### Many non-BS producing cells can hydrolyze LBS into BS

**Figure 2:**
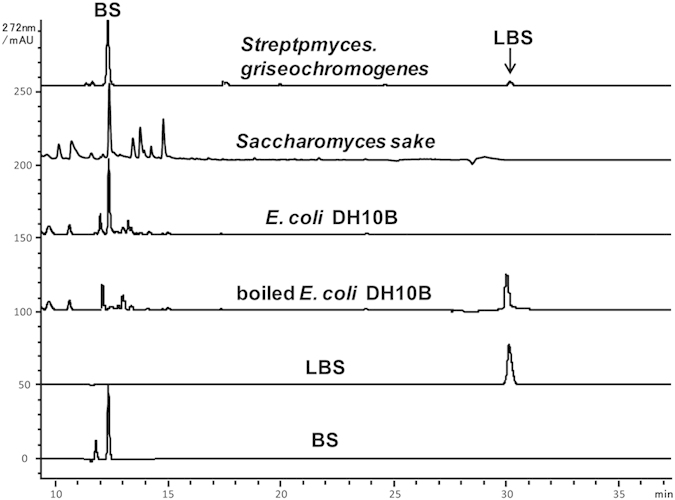
HPLC analysis of the reaction product by incubating LBS with the cell free extracts (CFE) of *Streptomyces griseochromogenes*, *Saccharomyces sake*, *E. coli* DH10B. All of them were found to convert LBS into BS. Boiled CFE of DH10B was used as negative control.

The washed cells of *S. griseochromogenes*, the BS native producer, were reported to hydrolyze LBS into BS^22^. We repeated this experiment using the cells of *S. lividans* WJ2 (harboring the BS gene cluster) and its parent strain *S. lividans* HXY16 (without the BS gene cluster) as the negative control. Surprisingly, the cells of both strains are capable of hydrolyzing LBS to BS in similar effective way. We further found that cells from *E. coli* DH10B and *Saccharomyces sake* are also able to accomplish this process. The cell free extracts of these strains were all found to possess LBS hydrolytic activity as well (Fig. 2). However, the boiled CFE of *E. coli* DH10B cannot perform this process, demonstrating that LBS hydrolysis is not a spontaneous process and that there is a conserved hydrolase gene in charge of the maturation of LBS into active BS.

### Identification of PepN in *E. coli* and *Streptomyces lividans* as the peptidase involved in LBS hydrolysis

**Figure 3:**
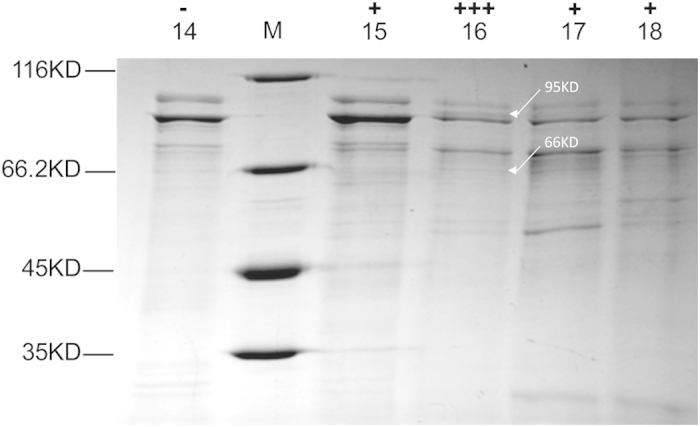
SDS-PAGE analysis of the protein fractions eluted from MonoQ HR 5/5 column. “−” represents this fraction has no LBS hydrolysis activity. “ + ” represents positive in LBS hydrolysis. There are some comparatively thicker bands in the most active No.16 fraction, such as bands of approximate 95 kD and 66 kD indicated by arrows.

As shown in Fig. 2, *E. coli*, the most studied bacterium, displayed relatively high efficiency in LBS hydrolysis and was thus chosen as the start strain to clone LBS hydrolase gene. The CFE of *E. coli* were precipitated with different concentration of ammonium sulfate wherein the fraction precipitated by 40% (V/V) ammonium sulfate displayed high catalytic activity, and further subjected to sequential separation on HiTrap Q HP (GE Healthcare, 5 ml) and on MonoQ HR 5/5 column (GE Healthcare). The eluate fractions from No.15 till No.19 showed LBS hydrolysis activity. These fractions and the inactive fraction No. 14 were analyzed by gel electrophoresis. There are some comparatively thicker bands in the most active No.16 fraction, such as bands of approximate 95 kD and 66 kD (indicated by arrows in Fig. 3). The fractions of No.14 and No.16 were analyzed by LC-MS/MS for their protein compositions (Supplementary Figure S2). 296 specific proteins were identified in the fraction of No.16 wherein the five most abundant peptidases were chosen as the candidates for the LBS hydrolase (Table 1).

**Figure 4:**
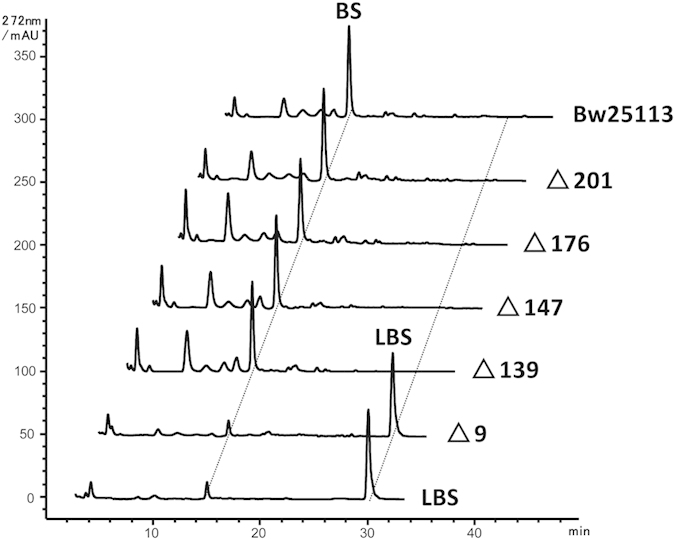
HPLC analysis of the reaction products for incubation of LBS with the CFEs of mutants of peptidase genes. Only the *pepN* (∆9) mutant lost all LBS hydrolytic activity while the other four mutants retained the same efficacy in LBS hydrolysis as the wild type.

The five peptidase genes were then disrupted in *E. coli* BW25113, an *E. coli* derivative facilitating homologous recombination between linear DNA fragment and chromosome. The CFE of the five mutants were assayed for LBS hydrolytic activity. Only the *pepN* mutant lost all LBS hydrolytic activity while the other four mutants retained the same efficacy in LBS hydrolysis as the wild type (Fig. 4). This result demonstrates that PepN is responsible for LBS hydrolysis in *E. coli*.

### *In vitro* activities of PepNs from *S. lividans* WJ2 and *E. coli* are affected by pH variation

*PepN* homologs are widespread in prokaryotes and eukaryotes and may function as the LBS hydrolase in various strains. Two closely located PepN homologs from *Streptomyces lividans* were identified through blast search; PepN1 is 27% identical (114/427) and 42% positive (180/427), and PepN2 is 26% identical (91/346) and 42% positive (146/346) to PepN, respectively. We thus expressed and purified PepN, PepN1 and PepN2 *in E. coli* BL21 (DE3) to assay their LBS hydrolysis activity (Left panel, Supplementary Figure.S3). PepN and PepN1 exhibited similar LBS hydrolyzing efficiency which is at least 100-fold higher than that for PepN2 (Right panel, Supplementary Figure S3). The transcription level of *pepN1* is comparable to that of *blsD* as well as constitutively expressed *hrdB*, but is at least 3 folds higher than that of *pepN2* in *S. lividans* WJ2 (Supplementary Figure S4), therefore PepN1 is the major LBS hydrolase in *S. lividans* WJ2 during fermentation.

**Figure 5:**
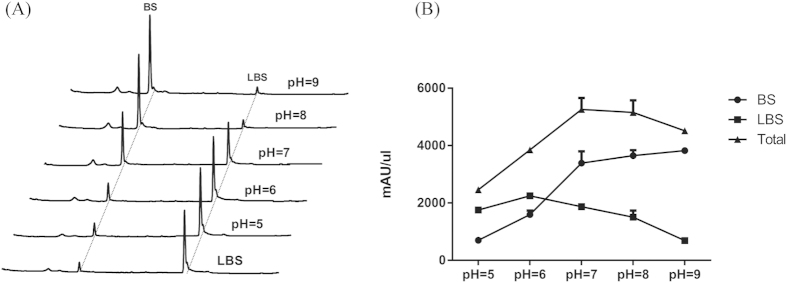
pH is the key factor in regulating PepN activity *in vitro* and *in vivo*. (**A)** HPLC analysis of reaction products for purified PepN1 with excessive LBS at pH ranging from 5 to 9 for 5 minutes, the most appropriate pH for *in vitro* reaction at 7.5 and least active above 10 or below 6, PepN activity is significantly affected by pH. (**B)** The production of BS and LBS and their total amount by *S. lividans* WJ2 when the pH of the fermentation medium was pre-adjusted to 5, 6, 7, 8 and 9, the pH is adjusted after medium sterilization.

As in different fermentations, the proportion of LBS in the final metabolites varies, implying that PepN activity is affected by the changing medium conditions, particularly the pH value as it was affected very much by cell contamination or sterilizations process. PepN and PepN1 were then measured for their activity in hydrolysis of LBS at pH ranging from 5 to 9. PepN and PepN1 behave similarly at the same pH, and the pattern change of activity are also similar, namely with most appropriate pH for *in vitro* reaction at 7.5 and least activity above 10 or below 5 (Fig. 5A). These observation are consistent with that pH variation in the fermentation broth affected the LBS hydrolase activity (Fig. 5B) and thus determined the proportion of LBS to BS in the final metabolites.

### Overexpression of PepN1 in *S. lividans* WJ2 to improve the yield of BS

**Figure 6:**
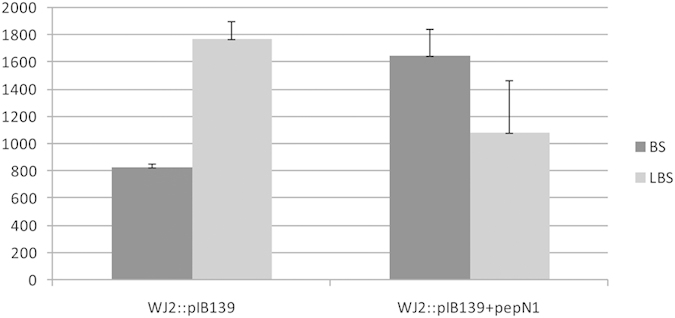
The yield of BS and LBS in *S. lividans* YGY7 (WJ2::pIB139), *S. lividans* YGY6 (WJ2::pIB139 + *pepN1*). The ratio of BS to LBS yield in the YGY6 is 3:2 as compared to that of 1:2 in YGY7, the yield of BS has nearly doubled.

As LBS is 20-fold less active than the final product BS^23^, *in vivo* conversion of LBS to BS by overexpression of *pepN1* in *S. lividans* WJ2 is desired. *pepN1* under the control of constitutive expression promoter PermE* were constructed and introduced into WJ2 to generate mutant strains *S. lividans* YGY6. The ratio of BS to LBS yield in the YGY6 is 3:2 as compared to that of 1:2 in WJ2::pIB139, the absolute yield of BS increased 100% upon the overexpression of PepN1(Fig. 6).These results demonstrated that overexpression of *pepN1* is an efficient way to enhance BS production.

## Discussion

Removal of the leucyl group from Leucylblasticidin S was proposed as the final step in BS biosynthesis^23^. Heterologous expression of blasticidin S biosynthetic gene cluster in *Streptomyces lividans* occasionally produces the mixture of LBS and BS. It remains a puzzle whether the LBS hydrolysis is a spontaneous process or the LBS hydrolase gene is included in the published BS biosynthetic gene cluster or locates elsewhere on the chromosome. In the process to clone the possible LBS hydrolase gene, we observed that CFE of some none-BS producers can efficiently covert LBS to BS whereas the boiled CFE of these strains cannot perform this process, demonstrating that LBS is not a spontaneous process and LBS hydrolase gene is outside of BS gene cluster. Hence, the published BS biosynthetic gene cluster actually encodes all essential proteins for LBS rather than BS. BS is now widely used commercially as a selection reagent in transgenic studies due to its higher inhibition activity than LBS, thus the study on the maturation mechanism for LBS into BS is of great application value.

We here report the identification of PepN as the LBS hydrolase by tracking the LBS hydrolytic activity in the CFE of *E. coli* and confirmed that PepN is the sole peptidase of *E. coli* to cleave the leucine from the beta-amino group of arginine. PepN in *E. coli* is a major aminopeptidase that is involved in downstream protein processing during cytosolic protein degradation and has a preference to cleave basic and small amino acids in an ATP-independent manner^26^. It is well conserved in all life kingdoms. Now that PepN1 and PepN2 were present and expressed in *Streptomyces lividans*, it is odd that WJ2 sometimes produces LBS at a ratio to BS as high as 9:1, but in some cases as low as 2:9, indicating that the fermentations conditions either affect the expression level of PepNs or the catalytic efficiency of PepNs in LBS hydrolysis. LBS was previously isolated when the fermentation broth was kept below pH 4 but later studies revealed it is unnecessary. This study again suggested that pH might plays an important role in regulation of the activity of PepNs. Consistent with this assessment is that the yield of BS is highest when the fermentation broth was kept at pH 8 as compared to lowest yield at pH 5. This finding provides a good way to promote the conversion of LBS into BS *in vivo* by simply adjusting the fermentation broth to pH 8. Given the above data, the possibility that pH plays a role in regulation of expression level of PepN *in vivo* could not be excluded.

An important concern on LBS hydrolysis is the location of where PepN acts on the substrate LBS. In the proposed pathway for BS biosynthesis, leucine is first loaded to the beta-amino group of L-arginine to form demethylleucylblasticidin S (LDBS), followed by methylation of the guanidino group to generate LBS. LDBS and LBS are both easily converted to DBS and BS by whole cells or the CFE of BS producer^23^. Thus, PepNs is unlikely to be co-localized with the enzyme forming LDBS, otherwise there could be a futile cycle of adding and removing leucine in the final steps of BS biosynthesis. Because analysis using HMMTOP (http://www.enzim.hu/hmmtop/index.php) revealed that PepNs has a short transmembrane region spanning from residues 384 to 401 and because, washed cells are capable of converting LBS and LDBS into BS and DBS, respectively, PepNs may be membrane-associated with its catalytic domain exposed to the cell exterior. The proposal is that LBS is formed within the cytoplasm either for self-protection or to facilitate export out of the cell where it is then hydrolyzed by PepNs that is anchored on the cell surface.

As PepN in *E. coli* is the major aminopeptidase that is responsible for the majority activity of aminopeptidase and is implicated in cytosolic protein degradation. This study showed that PepN is also capable of cleaving the amido bond between the carboxyl group of leucine and the beta-amino group of arginine that is bonded to the 4’-amino group of hexose ring of blasticidin S. It might also play a role in processing other secondary metabolites harboring moiety of amino acid or peptides.

## Methods

### Bacterial strains, plasmids and culture conditions

Strains, plasmids, and primers used in this study are listed in Supplementary Table S1 in the supplemental material. To purify the LBS hydrolase in the cell lysate, *Streptomyces* strains mycelia was cultured in 25 ml TSBY (tryptic soy broth [TSB] supplemented with 10.3% sucrose and 1% yeast extract, w/v) medium at 30 °C in 250-ml baffled flasks and at 220 rpm, and harvested after 36 hours incubation. For sporulation, *Streptomyces* strains were grown at 30 °C on SFM (soybean flour 2%, mannitol 2%, agar 1%, pH 7.2–7.4, w/v) agar plate for 5–7 days. SFM agar was also used for the conjugation between *Escherichia coli* and *Streptomyces lividans* WJ2. *E. coli* strains were grown at 37 °C in Luria Broth medium and on Luria Broth agar.

### Fermentation and HPLC analysis of blasticidin S and its derivatives

For blasticidin S and its intermediate metabolites analysis, the fermentation of strain *S. lividans* WJ2 and its derivatives was performed as previously described^11^. Cell broths were centrifuged at 10,000 × g for 10 min and the supernatants were applied to Supelclean LC-SCX solid-phase extraction (SPE) columns to remove unwanted non-BS derivatives. HPLC was operated on an Agilent series 1260 with a Thermo Golden C18 column (2.1 × 150 mm, 3 μm). The analytes were eluted at a flow rate of 0.2 ml/min with a gradient of 0% to 11% 20 mM ammonium acetate (pH 5.5) (buffer A) in the first 40 minutes and 95% acetonitrile (buffer B) in the following 5 minutes and were detected with UV absorption 272 nm.

### Preparation of cell-free extracts

Mycelia of *Streptomyces lividans* WJ2 or cells of *E. coli* and *Saccharomyces sake* were pelleted and washed twice in ten volumes of lysis buffer (50 mM K_3_PO_4_, pH 8.0, 1 mM dithiothreitol). The cells were then suspended in two volumes of lysis buffer in an ice bath and lysed by sonication. The cell lysate was centrifuged at 10,000 × g for 10 min at 4 °C, and the supernatant was either used for further purification or for *in vitro* enzymatic assays. The cell-free extract (CFE) was boiled in water for 10 min to inactivate the enzyme and then used as the negative control in the enzymatic assays.

### Ammonium sulfate precipitation and anion exchange column separation

CFE of *E. coli* was fractionated using 20%, 30%, 40%, and 50% ammonium sulfate (final concentration) respectively. The precipitates were collected and dissolved in dialysis buffer (50 mM NaCl, 50 mM Tris-Cl, pH 8.0), generating 20%, 30%, 40%, 50% (v/v) gradient protein components, respectively. These dissolved proteins were dialyzed against dialysis buffer overnight in a 4 °C cold room.

The most active protein fraction was further separated by a MonoQ HR 5/5 column (GE Healthcare) on the AKTA FPLC (GE Healthcare) system using a linear gradient of 50–500 mM NaCl. The elution was collected and the hydrolysis activity towards LBS was determined. LBS was prepared as reported in Zhang *et al.*^23^. The most active fraction and the one without LBS hydrolytic activity were compared on a 12% SDS-PAGE gel and sent for LC-MS/MS analysis (Shanghai BoYuan Biotechnology Co., LTD, China).

### Assay of the LBS hydrolytic activity of the CFE and protein eluates

1 μM LBS was incubated with 10 μl CFE or protein elutes in a total volume of 50 ul at 30 °C overnight (8–12 h), whereas in the control experiments boiled CFE was used. The reactions were quenched by adding 50 ul chloroform. The supernatant was analyzed by HPLC analysis as described above.

### PCR target disruption of the candidate genes encoding LBS hydrolase in *E. coli* BW25113 chromosome

PCR-targeted gene inactivation was achieved using REDIRECT^R^ Technology^27^. The pairs of primers Tar9-F/R, Tar139-F/R, Tar147-F/R, Tar176-F/R, Tar201-F/R with 5′-terminus paring with the putative hydrolase genes and 3′-terminus complementary to resistance cassette *aac(3)IV* (Supplementary Table S1) were used to generate linear fragments for targeted gene disruption. pIJ773 harboring *aac(3)IV* coding for apramycin resistance and FRT site-specific recombination sites was used as the template. 1.38 kb linear DNA fragments were generated and transformed separately by electroporation into *E. coli* BW25113. Mutants were selected by apramycin of 50 μg/ml final concentration and PCR verified using primers C-tar9F/R, C-tar139F/R, C-tar147F/R, C-tar176F/R, C-tar201F/R (Supplementary Table S1, Supplementary Figure S5).

### Heterologous expression and purification of PepN from *E. coli* and *S. lividans* WJ2

*pepN* genes from *E. coli* and *S. lividans* 1326 were amplified with primers PepN-F/R, PepN1-F/R and PepN2-F/R, respectively. They were sequenced and ligated into NdeI/XhoI-digested pET44b to generate pYGY1, pYGY2 and pYGY3 with C-terminal 6xHis tags. The recombinant proteins were expressed in *E. coli* BL21 (DE3) in LB medium supplied with 50 μg*/*ml ampicillin and 0.2 mM IPTG at 16 °C for 21 h. The recombinant proteins were purified to greater than 95% in purity determined by 12% SDS-PAGE using Ni-Sepharose™ 6 Fast Flow (GE). Protein concentrations were determined by Bradford Protein Assay Kit (Bio-Rad). LBS hydrolysis assays by purified PepN is the same as that described above for CFE assays.

### Overexpression of gene pepN1 in *S. lividans* WJ2

For overexpression of *pepN1* in *S. lividans* WJ2, *pepN1* was PCR amplified from *S. lividans* WJ2 with primer pair pepN1-139-F/R (Supplementary Table S1), and inserted into expression vector pIB139 between NdeI and XbaI under the control of constitutive promoter P_ermE*_. The recombinant plasmid pYGY4 and pIB139 were transformed into *E. coli* ET12567/pUZ8002 and transferred into *S. lividans* WJ2 by conjugation. The resultant mutants *S. lividans*::pYGY4 and *S. lividans*::pIB139 were cultured and compared in the LBS conversion rates with the wild type strain.

## Additional Information

**How to cite this article**: Yu, G. *et al.* The standalone aminopeptidase PepN catalyzes the maturation of blasticidin S from leucylblasticidin S. *Sci. Rep.* **5**, 17641; doi: 10.1038/srep17641 (2015).

## Supplementary Material

Supplementary Information

## Figures and Tables

**Table 1:** Five most abundant peptidases identified in fraction 16.

NO	Protein	Score	Mass	emPAI
9	Aminopeptidase N (PepN)	2311	99313	1.56
139	Predicted peptidase	244	45288	0.33
147	Predicted hydrolase	227	23200	0.71
176	Predicted hydrolase	172	34153	0.74
201	Predicted peptidase required for the maturation of the antibiotic peptide MccB17	138	48625	0.39

NO. represents the number of proteins in the order of decreasing score in fraction 16.

emPAI represents protein abundance.
