# Supplementary material for: The standalone aminopeptidase PepN catalyzes the maturation of blasticidin S from leucylblasticidin S
Source: Sci Rep. 2015 Dec 1;5:17641. doi: 10.1038/srep17641 (PMC4664946; doi:10.1038/srep17641)
Supplement: Supplementary Information [file srep17641-s1.doc]

Electronic Supplementary Materials for

The standalone aminopeptidase PepN catalyzes the maturation of blasticidin S from leucylblasticidin S

Guiyang Yu^1^, Li Li^2^, Xiangyang Liu^1^, Guang Liu^1^, Zixin Deng^1^, T. Mark Zabriskie^3^, Ming Jiang^1^* & Xinyi He^1^*

^1^State Key Laboratory of Microbial Metabolism and School of Life Science and Biotechnology, Shanghai Jiao Tong University, Shanghai 200030 (China)

^2^Engineering Research Center of Industrial Microbiology (Ministry of Education), and College of Life Sciences, Fujian Normal University, Fuzhou, Fujian 350108 (China)

^3^Department of Pharmaceutical Sciences, College of Pharmacy, Oregon State University, Corvallis, OR 97331-3507 (USA)

***For Correspondence** either to X.H. via [xyhe@sjtu.edu.cn](mailto:xyhe@sjtu.edu.cn)

or to M.J. via [Jiangming9722@sjtu.edu.cn](mailto:Jiangming9722@sjtu.edu.cn)

**Tel**: (86)-2162932943(EXT)

**Fax**: (86)-2162932418

Supplementary Materials

Table S1 ----- Reference ----- Method -----Figure S1-S5 & legends

Table S1 Strains, plasmids and primers used in this study.

| **Strains** | **Relevant properties** | **Source** |
| --- | --- | --- |
| *Streptomyces lividans* WJ2 | Blasticidin S heterologous expression  strain | 1 |
| *Streptomyces lividans* DAQ2 | Gene *blsN*-inactivated mutant | This study |
| *Streptomyces lividans* LL3 | Gene *bls-1*-inactivated mutant | This study |
| *Streptomyces lividans* LL4 | Gene *bls-2*-inactivated mutant | This study |
| *Streptomyces lividans* YGY6 | WJ2 with *pepN1*-overexpression | This study |
| *Streptomyces lividans* YGY7 | WJ2 with pIB139, negative control | This study |
| *Streptomyces griseochromogenes* | Producer of blasticidin S | CGMCG |
| *Saccharomyces sake* | Fungal  representative strain | This study |
| *Rhodotorula rubra* | Indicator strain of blasticidin S | 1 |
| *Escherichia coli* DH10B | F^-^ *rec*A *lac*Z △M15 | GIBCO BRL |
| *Escherichia coli* ET12567  /pUZ8002 | *rec*F, *dam, dcm, hsdS,* Cml^r^*,* Str^r^, Tet^r^, Km^r^ | 2 |
| *Escherichia coli* BL21 (DE3) | F^-^ ompT rB^-^ mB^-^ (λDE3) | Novagen |
| *Escherichia coli* BW25113/pIJ790 | RepA101(ts), *araBp-gam-be-exo*,  AraC, RepA101(ts) Cml^r^ | 2 |
| *Escherichia coli* YGY1 | BW25113 with ***9***  (aminopeptidaseN)-inactivated | This study |
| *Escherichia coli* YGY2 | BW25113 with ***139***(predicted  peptidase)-inactivated | This study |
| *Escherichia coli* YGY3 | BW25113 with ***147***(predicted  hydrolase)-inactivated | This study |
| *Escherichia coli* YGY4 | BW25113 with ***176***(predicted  hydrolase)-inactivated | This study |
| *Escherichia coli* YGY5 | BW25113 with ***201***(predicted  peptidase)-inactivated | This study |
| **Plasmids** |  |  |
| pET44b | Expression vector with 6XHis-tag. | Novagen |
| pIJ773 | *aac(3)IV* resistance cassette | 3 |
| pYGY1 | pET44b expressing *pepN* from *E. coli* | This study |
| pYGY2 | pET44b expressing *pepN1* from *S. lividans* | This study |
| pYGY3 | pET44b expressing *pepN2* from *S. lividans* | This study |
| pIB139 | *attP, Int, oriT, PermE*, aac(3)IV* | 4 |
| pYGY4 | pIB139 expressing *pepN1* | This study |
|  | | |
| Primers Sequences (from 5′ to 3′) | | |
| Tar9-F | CGGATTACCAGATTACTGATATTGACTTGACCTTTGACCATTCCGGGGATCCGTCGACC | |
| Tar9-R | TAGTTATCTTCTCGTACAGATCGCCAGAGAGATTTTCCATGTAGGCTGGAGCTGCTTC | |
| Tar139-F | AAAGATTACCAGGCAGACATGACTCGCTTCCTGCGCGATATTCCGGGGATCCGTCGACC | |
| Tar139-R | GCGGCACAGGTTACCAGGTGAGATTTCCAGGTTTTTTCGTGTAGGCTGGAGCTGCTTC | |
| Tar147-F | ATGCTGCCGTTTTGCTTGTTGATCACCAGGCTGGTTTACATTCCGGGGATCCGTCGACC | |
| Tar147-R | ACAACGTCGCCAGTCCTTCAATATCATTACGCCAGTCGCTGTAGGCTGGAGCTGCTTC | |
| Tar176-F | ATTGTGTTATCTCACCCAGGTGGGGGCGTTAAAGAACAAATTCCGGGGATCCGTCGACC | |
| Tar176-R | CAGCCACATAGGCTTTGCCGTCATAAAGATCCATATGGTTGTAGGCTGGAGCTGCTTC | |
| Tar201-F | CAAGATTCTGGAAGAAGCAGTTTCCACTGCGCTGGAGTTATTCCGGGGATCCGTCGACC | |
| Tar201-R | TCAATATCGTTACCGACGGTGACAATATTGCGCCACATATGTAGGCTGGAGCTGCTTC | |
| TarblsN-F | GTTCCGCCCGGCCGGGTGCTCCTGGATACTGGCTCAATGATTCCGGGGATCCGTCGACC | |
| TarblsN-R | TTCGCTCCGTAGGGCACGTTCCGTGCCGGATGCCCCTCATGTAGGCTGGAGCTGCTTC | |
| Tarbls-1-F | ATGGCCCACGTACTCGCCCTGACCGACGGCACCGCCTCCATTCCGGGGATCCGTCGACC | |
| Tarbls-1-R | CTACGGAGCGAAGACGCGGTCCAGCGCGGCCATGGTCCCTGTAGGCTGGAGCTGCTTC | |
| Tarbls-2-F | ATGAGTGCGCGGCAGCAGAACCCGTTTCTGGTCGGCAAGATTCCGGGGATCCGTCGACC | |
| Tarbls-2-R | TCATGCGAAGACGTCGAGTACGCCGTTCCGGTCGGAGTCTGTAGGCTGGAGCTGCTTC | |
| C-tar9F | ATGACTCAACAGCCACAAGC | |
| C-tar9R | AGCCAGTGCTTTAGTTATCT | |
| C-tar139F | GGCTAAGAATATTCCATTCAAACTG | |
| C-tar139R | CTTAATGGGATTGCAGCGTA | |
| C-tar147F | ATGACCAAACCGTATGTTCG | |
| C-tar147R | CGTGTCATAACTGGTCATCA | |
| C-tar176F | ATGAGGAACGGAATGATGAA | |
| C-tar176R | CGGAGCTAATACTGAAATTG | |
| C-tar201F | ATGGCACTTGCAATGAAAGT | |
| C-tar201R | CAGCACAGAACCACACTGTA | |
| C-tarblsNF | TTCTCGTCCGCGGTCTCGGTACCGT | |
| C-tarblsNR | ACCGGTCAGGCGGTGTCCGCCTTCA | |
| C-tarbls-1-F | ATGGCCCACGTACTCGCCCTG | |
| C-tarbls-1-R | CTACGGAGCGAAGACGCGGTC | |
| C-tarbls-2-F | ATGAGTGCGCGGCAGCAGAAC | |
| C-tarbls-2-R | TCATGCGAAGACGTCGAGTAC | |
| pepN-F | GGACcatatgACTCAACAGCCACAAGCCAA, NdeI site | |
| pepN-R | CCGctcgagAGCCAGTGCTTTAGTTATCT, XhoI site | |
| pepN1-F | GGACcatatgCCTGGCACAAACCTGACTCG, NdeI site | |
| pepN1-R | CCGctcgagCTCGGCCGCCGCCGCGTCCG, XhoI site | |
| pepN2-F | GGACcatatgCCCGGTGAGAATCTGTCCCGC, NdeI site | |
| pepN2-R | CCGctcgagCGCACCCCCGTCACAGGCCT, XhoI site | |
| pepN1-139-F | GGACcatatgCCTGGCACAAACCTGACTCGCGAA,  NdeI site | |
| pepN1-139-R | GCtctagaCTACTCGGCCGCCGCCGCGT, XbaI site | |
| C-pepN1-139F | TTCCGGAAGTGCTTGACATT | |
| C-pepN1-139R | ACCAGGTCGATGAAGGACTC | |
| R-blsD-F | GTGAGAACGTCAGTGCCGGA | |
| R-blsD-R | TTGGTGATCGAGGCGAGCAC | |
| R-pepN1-F | GTGCCTGGCACAAACCTGAC | |
| R-pepN1-R | TTCAGCGTCACCTCGTGGAC | |
| R-pepN2-F | AGGACGGCGAGGTGTACCTGTA | |
| R-pepN2-R | TGTACGTCGAGATCGGCTTC | |
| R-hrdB-F | GTCTCTGTCATGGCGCTCATT | |
| R-hrdB-R | CTTCGCTGCGACGCTCTTTC | |

**Reference**

1 Li, L., Wu, J., Deng, Z., Zabriskie, T. M. & He, X. Streptomyces lividans blasticidin S deaminase and its application in engineering a blasticidin S-producing strain for ease of genetic manipulation. *Applied and environmental microbiology* **79**, 2349-2357 (2013).

2 Kieser, T., Bibb, M. J., Chater, K. F., Butter, M. J. & Hopwood, D. A. Practical Streptomyces genetics. John Innes Foundation, United Kingdom, Norwich (2000).

3 Gust, B., Challis, G. L., Fowler, K., Kieser, T. & Chater, K. F. PCR-targeted Streptomyces gene replacement identifies a protein domain needed for biosynthesis of the sesquiterpene soil odor geosmin. *Proceedings of the National Academy of Sciences of the United States of America* **100**, 1541-1546, doi:10.1073/pnas.0337542100 (2003).

4 Wilkinson, C. J. *et al.* Increasing the efficiency of heterologous promoters in actinomycetes. *Journal of molecular microbiology and biotechnology* **4**, 417-426 (2002).

**Method**

*RNA isolation and reverse transcription PCR (RT-PCR).*

For RT-PCR experiments, the *S. lividans* WJ2 strain were grown in fermentation medium. After 24h and 48h, 1 ml cell culture were harvested respectively and washed twice with sterile water. For total RNA isolation, the cell pellet was resuspended with 1 ml Redzol reagent (sbs) by pipetting. Cells were disrupted using glass beads (100 µm, BioSpec) and a Precellys homogenizer (6500 rpm 20–30 s). RNA isolation was performed with an RNA extraction kit (sbs). RNA concentrations and quality were checked using a NanoDrop ND-2000 spectrophotometer (Thermo Fisher Scientific). 5µg RNA samples were treated with DNase (Fermentas) for 4 h at 37 °C. No DNA contamination was verified by PCR. 5 µg RNA was used to generate cDNA using random hexamer primers, cofactors and reverse transcriptase (Fermentas). As an internal control, the major sigma factor ( hrdB) transcript, which is produced constitutively, was amplified for each sample. All the primers were listed in supplementary Table S1.

**Figures**


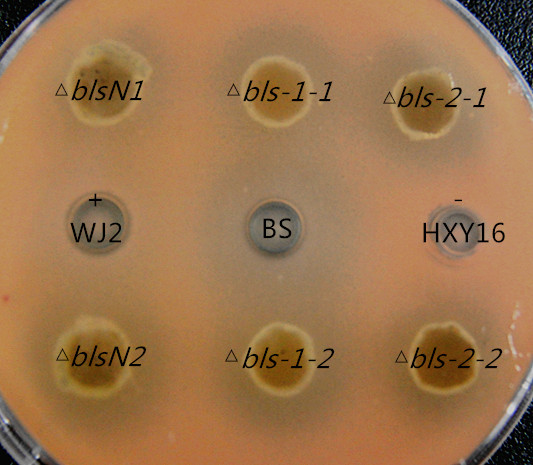

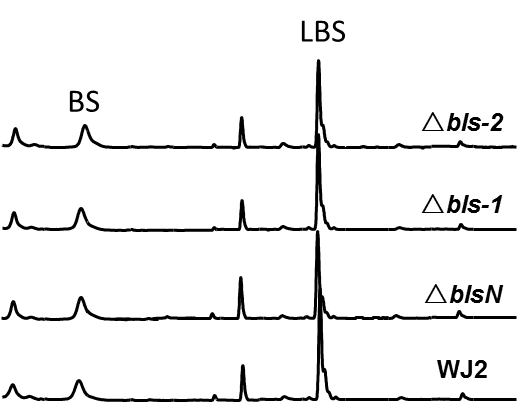
**(A) (B)**

Figure S1 Metabolites and biological activity of three mutants revealed no difference from strain *S. lividans* WJ2. (A) HPLC analysis of the metabolite production of *S. lividans* WJ2, *△blsN*, *△bls-1*, *△bls-2*. (B) Bioassay of *S. lividans* WJ2, BS standard, *S. lividans* HXY16, *△blsN*, *△bls-1*, *△bls-2*.

**
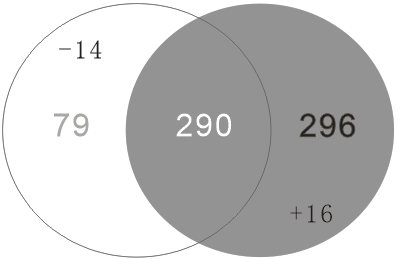
**

Figure S2 Protein components of fraction 14 and 16 were determined by LC-MS/MS assay. **79** and **296** represents the number of fraction-specific proteins. **290** represent overlapping proteins.

**(A)**

**
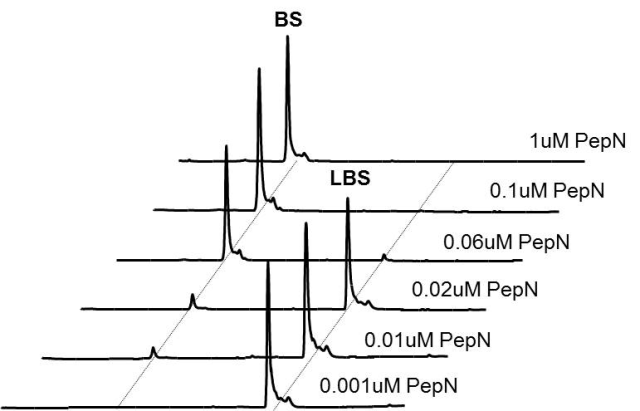

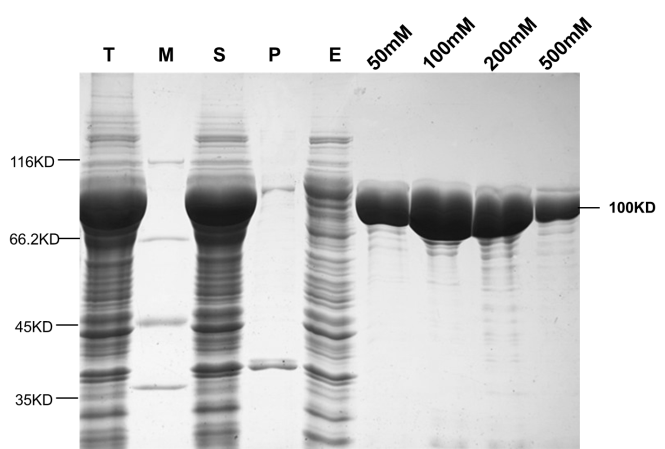
**


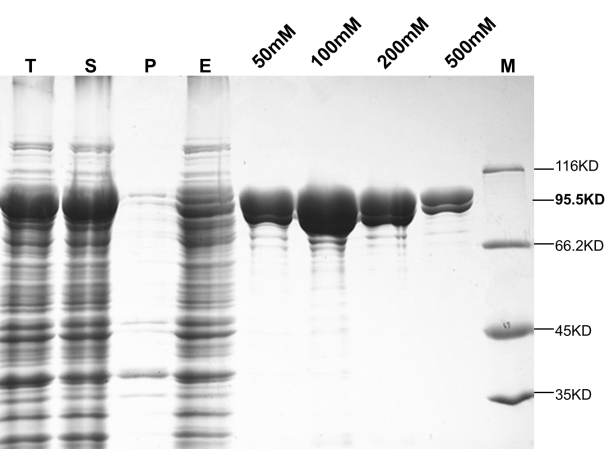

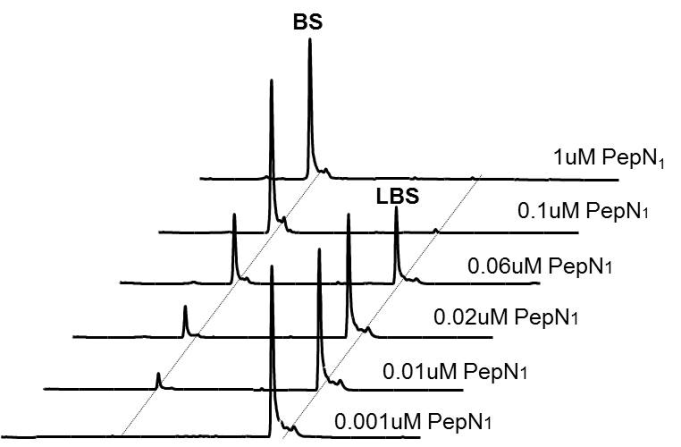
**(B)**

**(C)**

**
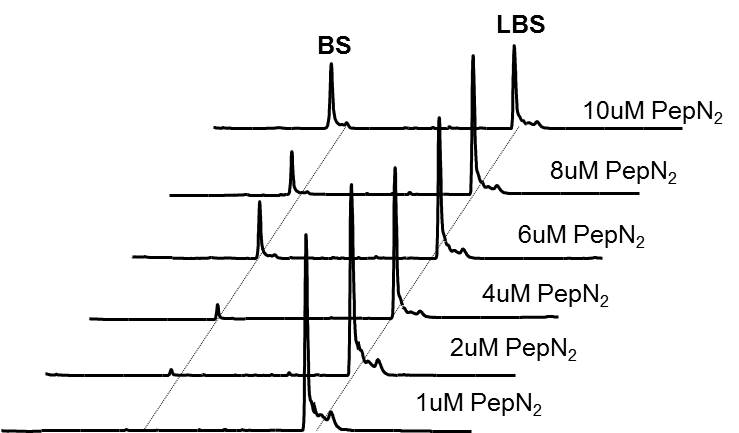

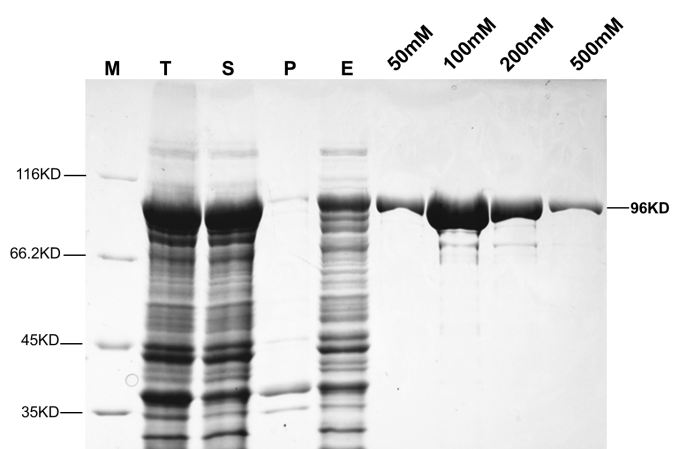
**

Figure S3 SDS-PAGE analysis of purified PepN, PepN1, PepN2 (A, B, C, Left panel). PepN and PepN1 exhibited similar LBS hydrolyzing efficiency which is at least 100-fold higher than that for PepN2 (Right panel).


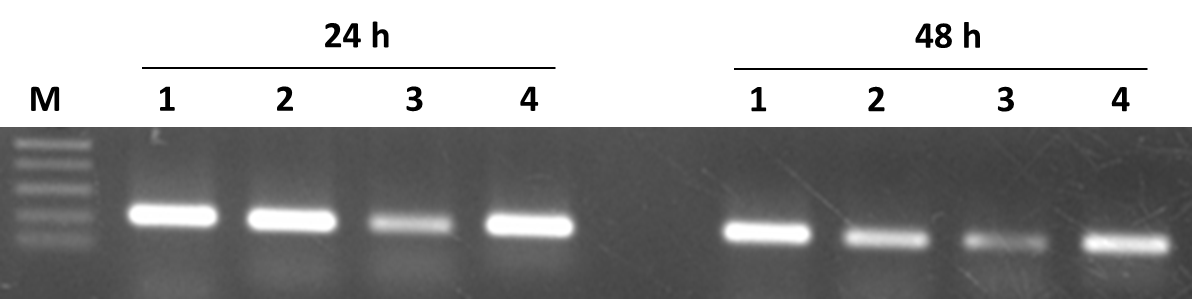


Figure S4 Transcriptional analysis of *blsD* (1), *pepN1* (2), *pepN2* (3) and *hrdB* (4) at 24 h (left panel) and 48 h (right panel) in *S. lividans* WJ2. *blsD*, encoding the cytosylglucuronic acid synthase in BS biosynthesis was used as inner control; hrdB, encoding the Sigma-70 factor as a general control.


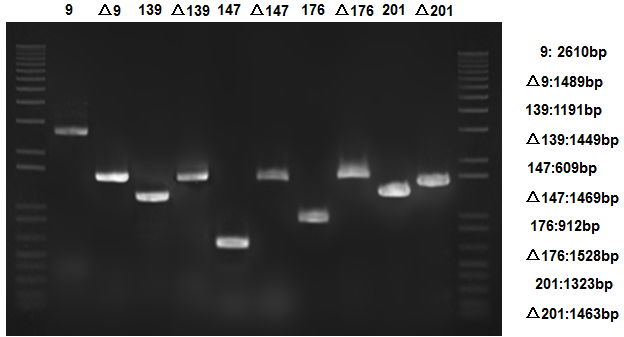


Figure S5 Confirmation of the mutants of five peptidase genes by PCR. The size of PCR products corresponding target gene in wild type and mutant is shown on the right side of gel picture.
